# Whole genome sequencing of *Salmonella enterica* serovars isolated from humans, animals, and the environment in Lagos, Nigeria

**DOI:** 10.1186/s12866-023-02901-1

**Published:** 2023-06-13

**Authors:** Kabiru Olusegun Akinyemi, Christopher Oladimeji Fakorede, Jörg Linde, Ulrich Methner, Gamal Wareth, Herbert Tomaso, Heinrich Neubauer

**Affiliations:** 1 Department of Microbiology, Faculty of Science, Lagos State University, Ojo, Lagos Nigeria; 2 Institute of Bacterial Infections and Zoonoses, Friedrich-Loeffler-Institute, Jena, Germany; 3 Department of Bacteriology, Immunology, and Mycology, Faculty of Veterinary Medicine, Benha University, PO Box 13736, Toukh, Moshtohor, Egypt; 4 Institute of Infectious Diseases and Infection Control, Jena University Hospital, Am Klinikum 1, 07747 Jena, Germany

**Keywords:** *Salmonella*, WGS, MLST, Virulence gene, Resistant gene, Serotyping, Nigeria

## Abstract

**Background:**

*Salmonella* infections remain an important public health issue worldwide. Some serovars of non-typhoidal *Salmonella* (NTS) have been associated with bloodstream infections and gastroenteritis, especially in children in Sub-Saharan Africa with circulating *S. enterica* serovars with drug resistance and virulence genes. This study identified and verified the clonal relationship of Nigerian NTS strains isolated from humans, animals, and the environment.

**Methods:**

In total, 2,522 samples were collected from patients, animals (cattle and poultry), and environmental sources between December 2017 and May 2019. The samples were subjected to a standard microbiological investigation. All the isolates were identified using Microbact 24E, and MALDI-TOF MS. The isolates were serotyped using the Kauffmann-White scheme. Antibiotic susceptibility testing was conducted using the disc diffusion method and the Vitek 2 compact system. Virulence and antimicrobial resistance genes, sequence type, and cluster analysis were investigated using WGS data.

**Results:**

Forty-eight (48) NTS isolates (1.9%) were obtained. The prevalence of NTS from clinical sources was 0.9%, while 4% was recorded for animal sources. The serovars identified were *S*. Cotham (n = 17), *S*. Give (n = 16), *S*. Mokola (n = 6), *S.* Abony (n = 4), *S*. Typhimurium (n = 4), and *S*. Senftenberg (n = 1). All 48 *Salmonella* isolates carried intrinsic and acquired resistant genes such as aac.6…Iaa, mdf(A), qnrB, qnrB19 genes and golT, golS, pcoA, and silP, mediated by plasmid Col440I_1, incFIB.B and incFII. Between 100 and 118 virulence gene markers distributed across several *Salmonella* pathogenicity islands (SPIs), clusters, prophages, and plasmid operons were found in each isolate. WGS revealed that strains of each *Salmonella* serovar could be assigned to a single 7-gene MLST cluster, and strains within the clusters were identical strains and closely related as defined by the 0 and 10 cgSNPs and likely shared a common ancestor. The dominant sequence types were *S.* Give ST516 and *S.* Cotham ST617.

**Conclusion:**

We found identical *Salmonella* sequence types in human, animal, and environmental samples in the same locality, which demonstrates the great potential of the applied tools to trace back outbreak strains. Strategies to control and prevent the spread of NTS in the context of one’s health are essential to prevent possible outbreaks.

**Supplementary Information:**

The online version contains supplementary material available at 10.1186/s12866-023-02901-1.

## Background

Salmonellosis in humans is caused by Gram-negative zoonotic bacteria of the species *Salmonella enterica* and *Salmonella bongori* and remains an important public health problem worldwide. Salmonellosis accounts for 93.8 million cases of gastroenteritis, with an estimated 155,000 deaths recorded globally every single year [1]. Most cases of salmonellosis are acquired from contaminated food such as dairy and poultry products [2]. Of the more than 2,600 different serovars of *S. enterica*, only a few non-typhoidal serovars (NTS) are responsible for most human infections [2]. *Salmonella* Typhi and *S*. Paratyphi, which are human-restricted, cause systemic illness, i.e., typhoid or paratyphoid fever [3]. NTS serovars are diverse in their host range and vary in their pathogenicity [4]. For severe cases of salmonellosis like sepsis or SIRS (Systemic Inflammatory Response Syndrome), antibiotic treatment is indicated. Unfortunately, multidrug resistance (MDR) with resistance to ampicillin, chloramphenicol, and trimethoprim-sulfamethoxazole has become a growing concern in NTS and some invasive non-typhoidal *Salmonella* (iNTS) infections [5]. Fluoroquinolone resistance in isolates from African countries has increased in recent decades, and resistance to β-lactam antibiotics, particularly third- and fourth generation cephalosporins, was found in Nigeria [6]. The development of NTS isolates resistant to extended spectrum cephalosporins such as ceftriaxone represents another substantial public health issue [7]. In Africa, NTS strains appear to be different from those that cause diarrheal disease in industrialized countries and cause invasive disease with bacteraemia more often in children, with 4100 deaths per year [8, 9]. The role of animal reservoirs and human-to-human transmission of iNTS strains is unclear [10, 11]. The control of zoonotic diseases in Nigeria is difficult due to the diversity of reservoirs and lack of surveillance. Zoonotic pathogens can contaminate the environment, spill over into the food chain, and appear at any time during processing and post-processing procedures. 29% of the national burden of human disease has been linked to the environment in Nigeria, while the remaining 71% has been traced to diarrheal diseases, malaria, respiratory infections, etc. [11]. A high prevalence of *Salmonella* in commercial poultry farms (43.6%) was reported [12]. Products from cattle and poultry have been identified as major sources of human *Salmonella* infections in Nigeria [13]. Furthermore, poultry and poultry products are the major sources of protein for humans. The domestic industry (Poultry Industry) rose from an estimated 350,000 metric tons (MT) of eggs and 200,000 MT of poultry meat produced in 2003 to an estimated production of 650,000 MT of eggs and 290,000 MT of poultry meat in 2013 [14, 15]. The industry is estimated to be worth $600 million, i.e., approximately 165 million birds in 2013. By 2015, these numbers had risen to about 180 million birds, with the bulk of the poultry production coming from backyard poultry farming. This increase is attributed to the ban on the import of chicken (excluding day-old chicks) in 2003 [14, 15]. A recent survey (2008–2015) on *Salmonella* bacteraemia in children in central and northwest Nigeria revealed that 20.7–23.6% of the *Salmonella* bacteraemia cases were due to non-typhoidal *Salmonella*, with up to 45% and 39% of the isolates being *Salmonella* Typhimurium and *S.* Enteritidis, respectively [16]. Bacterial resistance to antibiotics can be attributed to irrational use due to advertising strategies, application without previous susceptibility testing, or consumption of food produced from previously treated animals [17]. Antimicrobial resistance due to the acquisition of resistance gene clusters either horizontally or vertically is on the rise [18]. These gene clusters have been reported to have an epidemiological link with community-associated infections in many countries, including Nigeria [18–20]. The benefit of the recent explosion of genomic sequence information for *Salmonella* to study drug resistance in *Salmonella* serotypes cannot be overemphasized. In outbreak investigations and epidemiological surveillance, many laboratories, including reference laboratories, have employed serotyping and phage typing for decades [21]. But due to the polyphyletic nature of *Salmonella* serovars, evolutionary groupings may fail if phenotypic methods such as serotyping are used alone [21]. Thus, in the last two decades, pathogen characterization has already shifted to genomic analysis techniques, e.g., multi-locus sequence typing (MLST) and the variable number of tandem repeats (VNTR) [22, 23]. Since whole-genome sequencing (WGS) has become widely available and affordable as a tool for genotyping bacteria, it is replacing older genomic techniques [24]. The *Salmonella* Typhi genome was first reported in 2001, and several thousand *Salmonella* strains of various serovars have been sequenced [7, 24]. The challenge of discriminating highly related lineages of bacteria was resolved by analysis of the genome sequence using WGS and bioinformatics pipelines [24, 25]. In Nigeria, there are currently few studies on the molecular detection of virulence and antimicrobial resistance genes [26, 27].

The aim of this study is to investigate virulence and antimicrobial resistance genes among the *Salmonella enterica* serovars and to identify potential clonal relationships between strains from different sources using whole genome sequencing.

## Materials and methods

### Ethical approvals

Ethical approval from the ethics committee of the following institutions was obtained before patients’ enrolment: The Human Research and Ethics Committee of the Lagos State University Teaching Hospital with reference number LREC/06/10/1012 and the Lagos State Health Service Commission with reference number LSHSC/2222/VOL.VC/352.

### Preliminary investigation

A total of 2,522 samples were collected between December 2017 and January 2019, comprising 2,002 human samples (blood 1,042 and stool 960) from hospitalized and outpatients with clinical diagnoses of febrile illness and diarrheal disease, 150 samples of cattle dung (animal from the point of slaughter) and 270 samples of poultry feces (birds ready for sale), and 100 hospital effluent (wastewater) samples. Samples were screened for *Salmonella* growth using standard protocols [28, 29] at the Department of Microbiology, Lagos State University, Nigeria.

### Bacteria identification

A total of 51 presumptive *Salmonella* isolates, comprising 13 strains from in-patients of the general hospitals (Alimosho General Hospital and Massy Street Children Hospital), 9 strains from out-patients of the Lagos State University Teaching Hospital, 11 and 6 strains from dung and faecal samples of cattle and poultry respectively, and 12 strains from wastewater of Gbagada General Hospital, were initially identified using a MICROBACT 24E identification system (Oxoid Ltd, Basingstoke, UK). The isolates were stored in cryotubes with Nutrient-Agar (Oxoid Ltd., Basingstoke, UK) at room temperature and sent to the Institute of Bacterial Infections and Zoonoses (IBIZ) of the Friedrich-Loeffler-Institut (FLI) in Jena, Germany. These isolates were further analyzed following DIN EN ISO 6579-1:2017-07. Briefly, they were re-suspended in 3 mL of buffered peptone water for 6–18 ± 2 h (Oxoid Ltd., Basingstoke, UK) at 37 °C. Enrichment in a selective liquid medium was done in Rappaport-Vassiliadis (RVS) Soya Peptone Broth (RVS Broth) (OMNILAB-Laborzentrum GmbH, Bremen, Germany) for 24 ± 3 h at 41.5 °C. For selective media cultivation, two methods according to DIN EN ISO 6579-1:2017-07 were applied. Animal isolates were cultured on Xylose-Lysin-Deoxycholate Agar (XLD) (Oxoid Ltd., Basingstoke, UK) and Rambach Agar (Merck KGaA, Darmstadt, Germany) at 37 °C for 24 ± 3 h. Human and sewage isolates were cultivated on Rambach Agar and Bismuth Sulfite Agar (Becton Dickinson, Franklin Lakes, USA) at 37 °C for 24 ± 3 h for the early screening and detection of typhoidal and paratyphoid *Salmonella*.

For species confirmation of the animal isolates, an Ultraflex II MALDI-TOF MS instrument (Bruker Daltonik GmbH, Bremen, Germany) was used as described by [30]. After positive *Salmonella* confirmation, serological testing was done according to ISO/TR 6579-3:2014 for serovar identification.

The early identification of typhoidal and paratyphoidal *Salmonella* serovars for the human and sewage isolates was done with serological tests according to ISO/TR 6579-3:2014. The species confirmation for *Salmonella* isolates was done with anti-*Salmonella* A-67 + Vi omnivalent (Sifin Diagnostics GmbH, Berlin, Germany). Then, the O groups for *S.* Typhi (O: 9), *S.* Paratyphi A (O: 2), *S.* Paratyphi B (O: 4), and *S.* Paratyphi C (O: 7) were tested. A BSL-3 laboratory was available to further characterize isolates that would be found positive for anti-*Salmonella* O: 9 (Sifin Diagnostics GmbH, Berlin, Germany) and therefore suspicious to be *S.* Typhi. The non-suspicious isolates were also identified using an Ultraflex II MALDI-TOF MS instrument as *Salmonella* at the genus level (Bruker Daltonik GmbH, Bremen, Germany). Analysis was carried out with the Biotyper 3.1 software (Bruker Daltonik GmbH).

#### MALDI-TOF MS

Isolates were identified using MALDI-TOF MS [30]. Briefly, bacteria from overnight cultures were suspended in 300 µl of bi-distilled water and mixed with 900 µl of 96% ethanol (Carl Roth GmbH, Karlsruhe, Germany) for precipitation. After centrifugation for 5 min at 10,000 x g, the supernatant was removed, and the pellet was re-suspended in 50 µl of 70% (vol/vol) formic acid (Sigma-Aldrich Chemie GmbH, Steinheim, Germany). Fifty microliters of acetonitrile (Carl Roth GmbH) were added, mixed, and centrifuged for 5 min at 10,000 x g. One and a half microliters of the supernatant were transferred onto an MTP 384 Target Plate Polished Steel TF (Bruker Daltonik GmbH, Bremen, Germany). After air-drying, the material was overlaid with 2 µl of a saturated solution of -cyano-4-hydroxycinnamic acid (Sigma-Aldrich Chemie GmbH) in a mix of 50% acetonitrile and 2.5% trifluoroacetic acid (Sigma-Aldrich Chemie GmbH). After air-drying, spectra were acquired with an Ultraflex II instrument (Bruker Daltonik GmbH). The instrument was calibrated using the IVD Bacterial Test Standard (Bruker Daltonik GmbH). Analysis was carried out with the Biotyper 3.1 software (Bruker Daltonik GmbH). The following interpretation of results was performed according to the manufacturer’s recommendation: A score of ≥ 2.3 represented reliable species-level identification; a score of 2.0–2.29 represented probable species level identification; a score of 1.7–1.9 represented probable genus-level identification; and a score ≤ 1.7 was considered an unreliable identification [31]. Pure cultures were stored appropriately in cryo-tubes at -80 °C (Mast Diagnostica GmbH, Reinfeld, Germany).

### **Serotyping using the traditional White-Kaufman Le-Minor serotyping of 48*Salmonella*isolates**

All *Salmonella* strains were serotyped using poly- and monovalent anti-O as well as anti-H sera (SIFIN, Germany) according to the Kauffmann-White scheme [32].

### Antibiotic resistance testing

The isolates were re-cultivated on Columbia agar plates with 5% sheep blood for 24 h at 37 °C for antibiotic susceptibility testing with the Vitek 2 Compact system (BioMérieux, Marcy-etoile, France) according to EUCAST guidelines [33] for 24 different antibiotics on two cards (AST-N195 and AST-N248): ampicillin, amoxicillin + clavulanic acid, piperacillin, piperacillin-tazobactam, cefalexin, cefuroxime, cefuroxime-acetyl, cefotaxime, ceftazidime, cefepime, azithronam, ertapenem, imipenem, meropenem, amikacin, gentamicin, Tobramycin, ciprofloxacin, tigecycline, fosfomycin, nitrofurantoin, colistin, trimethoprim, and trimethoprim-sulfamethoxazole. Appropriate dilutions of the colonies were made according to the manufacturer’s instructions for MIC evaluation. The strains with Vitek Extended Spectrum ß-Lactamase (ESBL) were further characterized phenotypically: ESBL (CTX-M like), AmpC (High-Level Case (AmpC)), or/and carbapenemase (Carbapenemase (+ Oder - ESBL) phenotype were confirmed using a combination disk test (CDT) according to the manufacturer’s instructions and EUCAST guidelines. For ESBL resistance testing, MASTDISCS® Combi Extended Spectrum ß lactamase (ESBL) Set (CPD10) (Mast Diagnostica GmbH, Reinfeld, Germany) with Ceftazidime 30 µg and Ceftazidime 30 µg + Clavulanic Acid 10 µg, Cefotaxime 30 µg, and Cefotaxime 10 µg + Clavulanic Acid 10 and cefpodoxime 30 µg and Cefpodoxime 10 µg + Clavulanic Acid 1 µg were used. For the detection of carbapenemases, KPC, MBL, and OXA-48 MASTDISCS® were used: carbapenemases (Rosco, Taastrup, Denmark) with Meropenem 10 µg, Meropenem 10 µg + Phenylboronic acid, Meropenem 10 µg + Dipicolinic acid, Meropenem 10 µg + Cloxacillin, and Temocillin. For the AmpC detection, the 69 C AmpC Detection Disc Set (Mast Diagnostica GmbH, Reinfeld, Germany) was used with cefpodooxime 10 µg, AmpC stimulator, an ESBL inhibitor, and an AmpC inhibitor.

### Whole genomic sequence and bioinformatics analysis

#### Next-generation-sequencing (NGS)

The QIAGEN® Genomic-tip 20/G kit (QIAGEN, Germany) was used to prepare genomic DNA. NGS libraries were prepared using the NextEra XT DNA Library Preparation Kit (Illumina Inc., USA). An Illumina MiSeq instrument (Illumina Inc., USA) was used for paired-end sequencing. Raw sequences from this study are available and were deposited in the European Nucleotide Archive (ENA) with bio project accession PRJEB56537 in the ENA bio project database: https://www.ebi.ac.uk/ena/browser/view/PRJEB56537.

### Bioinformatics analysis: data analysis

The Linux-based bioinformatics pipeline WGSBAC v. 2.2.0 (https://gitlab.com/FLI_Bioinfo/WGSBAC) was used to analyze raw sequencing data as previously described [34, 35]. For quality control, WGSBAC used FastQC v. 0.11.7 [36] and calculated sequencing coverage. As a next step, the pipeline assembled sequencing reads using Shovill v. 1.0.4 [37] and accessed assembly quality using QUAST v. 5.0 [38]. To check for potential contamination, Kraken v2.1.1 [39] was used to classify the raw sequencing. To predict serovars based on sequencing data, WGSBAC used SISTR v. 1.0.2 [40] and SeqSero2 [41].

For the detection of genes and point mutations potentially leading to antimicrobial resistance (AMR), AMRFinderPlus (v. 3.6.10) [42] was used within WGSBAC. ABRicate (v. 0.8.10) [43] together with the databases Virulence Factor Database (VFDB) [44] and PlasmidFinder [45] were used to detect virulence factors and plasmids, respectively. For genotyping, WGSBAC first performed 7-gene multi-locus sequence typing (MLST) on assembled genomes using the software mlst v. 2.16.1 [46]. High-resolution genotyping was performed using both an SNP-based approach and an allele-based approach. Snippy v. 4.3.6 to identify core-genome single nucleotide polymorphisms (cgSNPs) was utilized within WGSBAC in standard settings [47]. As a reference genome, the complete genome sequence of *Salmonella enterica subsp. enterica serovar Typhimurium strain LT2 (GenBank accession GCA_000006945.2)* was used. To calculate pairwise SNP distances, SNPs-dists (v 0.63) were applied. Hierarchical clustering was performed using the hierClust function v.5.1 of the statistical language R. A cut-off of 10 cgSNPs was used to define closely related strains and 0 cgSNPs to define identical strains.

For the allele-based approach, core-genome multi-locus sequence typing (cgMLST) was performed by applying Ridom Seqsphere + v. 5.1.0 [48] with default settings together with the specific core-genome scheme (cgMLST v2) for *Salmonella enterica* developed by EnteroBase [49]. Again, a cut-off of 10 alleles was used to define clusters.

## Results

### **Identification, distribution, and serotyping of Nigerian*Salmonella enterica *isolates**

**Fig. 1:**
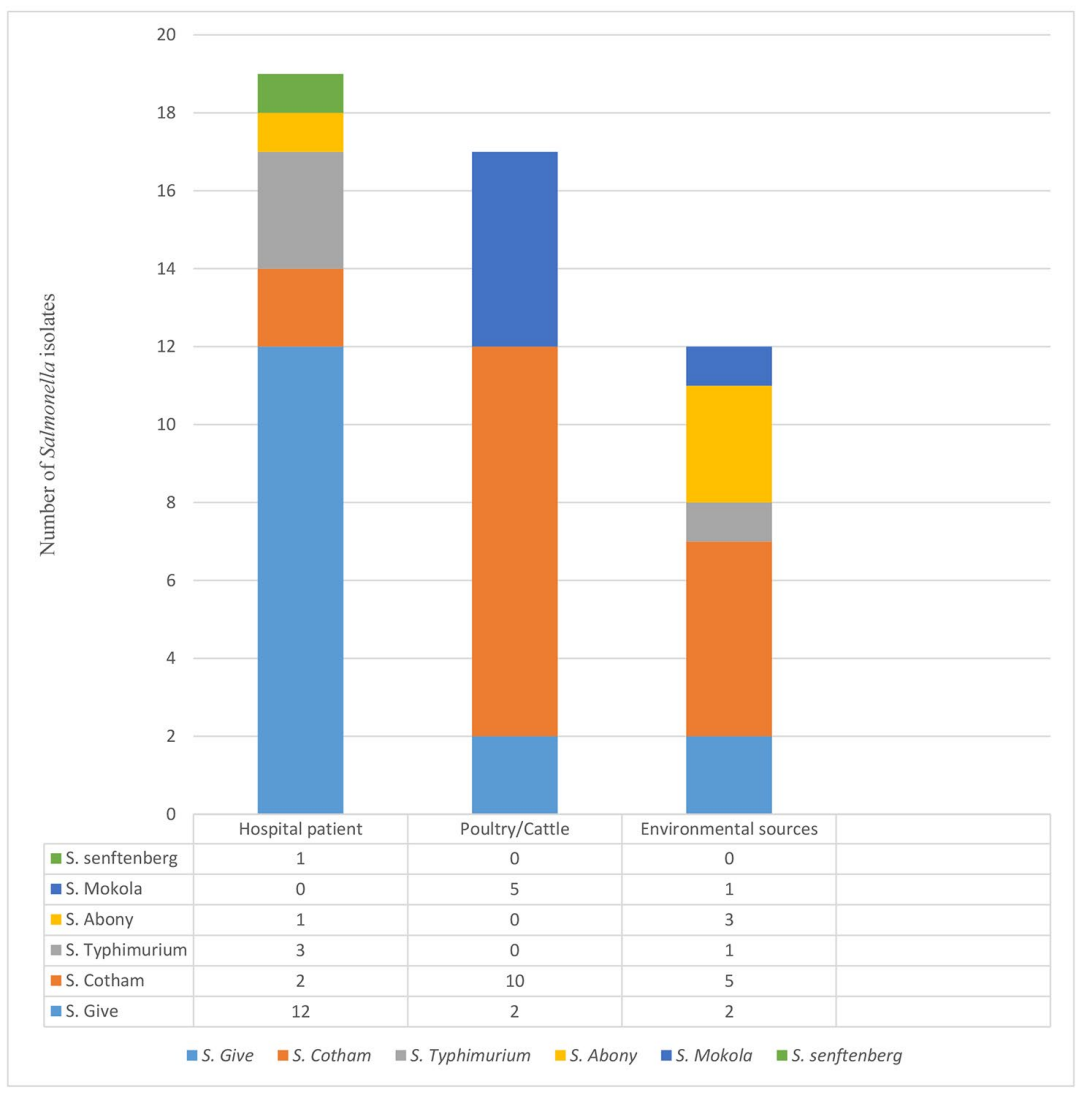
Distribution of *Salmonella enterica* serovars according to sample source from Lagos, Nigeria

Out of 2,522 samples analyzed in this study, 667 samples showed bacterial growth on selective media. From these positive bacterial culture samples, 51 presumptive *Salmonella* isolates were identified by Microbact 24E (Oxoid, England). Of the 51 presumptive *Salmonella* isolates, 48 isolates were identified as *Salmonella enterica* using MALDI-TOF MS **(**Table 1**)**, and the remaining three isolates were *Citrobacter* spp. The serotyping results of the 48 *Salmonella* isolates revealed six different serovars from human, animal, and environmental sources **(**Fig. 1**).** The serovars with their predicted antigenic profiles included *S.* Cotham (n = 17), *S. Give* (n = 16), *S.* Mokola (n = 6), *S.* Abony (n = 4), *S.* Typhimurium (n = 4) and *S.* Senftenberg (n = 1) and are presented in Table S1. The distribution of the serovars is as follows: *S*. Cotham (2 from human, 10 from animal, and 5 from sewage samples), *S*. Give (12 from human, 2 from animal, and 2 from sewage samples), *S.* Mokola (5 from animal samples and 1 from a sewage sample), *S.* Abony (1 from a clinical sample and 3 from sewage samples), *S.* Typhimurium (3 from human samples and 1 from a sewage sample), and *S.* Senftenberg ( 1 from a human sample), In this study, seven strains of S. Give, one strain of S. Typhimurium, and one strain of S. Senftenberg were iNTS.

**Table 1:** Sample summary and *Salmonella enterica* positive isolates distributed across human, animal and environmental sources

	Human samples		Animal samples Cattle Poultry		Environmental samples	Total
	Blood	Stool	Dung	Feaces	Effluent	
**Number of samples**	1042	960	150	270	100	2,522
**Number of + ve cultures**	163	308	54	72	70	667
***Salmonella*** **isolates (n)**	10	9	11	6	12	48
**Other isolates**	153	299	43	66	58	619

*+ve: positive bacterial culture

### Whole genome sequencing data

Genome sequencing of the 48 *Salmonella enterica* isolates analyzed in this study yielded an average of 1,513,603 reads per isolate (range 465,448-3,046,296; Table S1). The mean coverage of the 48 *Salmonella* isolates was 52-fold (ranging from 23-fold to 148-fold in **Table S2**). To check for putative contamination, the software Kraken2 was used, which classified each read (or contig). On the species level, the top hit for all 48 isolates was always “Salmonella”. On average, 96% of the reads were classified as “Salmonella”. **Table S3**. The N50 of the 48 assembled genomes ranges from 153,458 bp to 708,946 bp. (**Table S4)**

### **Resistance profiling and AMR genes in 48 Nigerian *Salmonella enterica *serovar isolates from different sources**

**Fig. 2:**
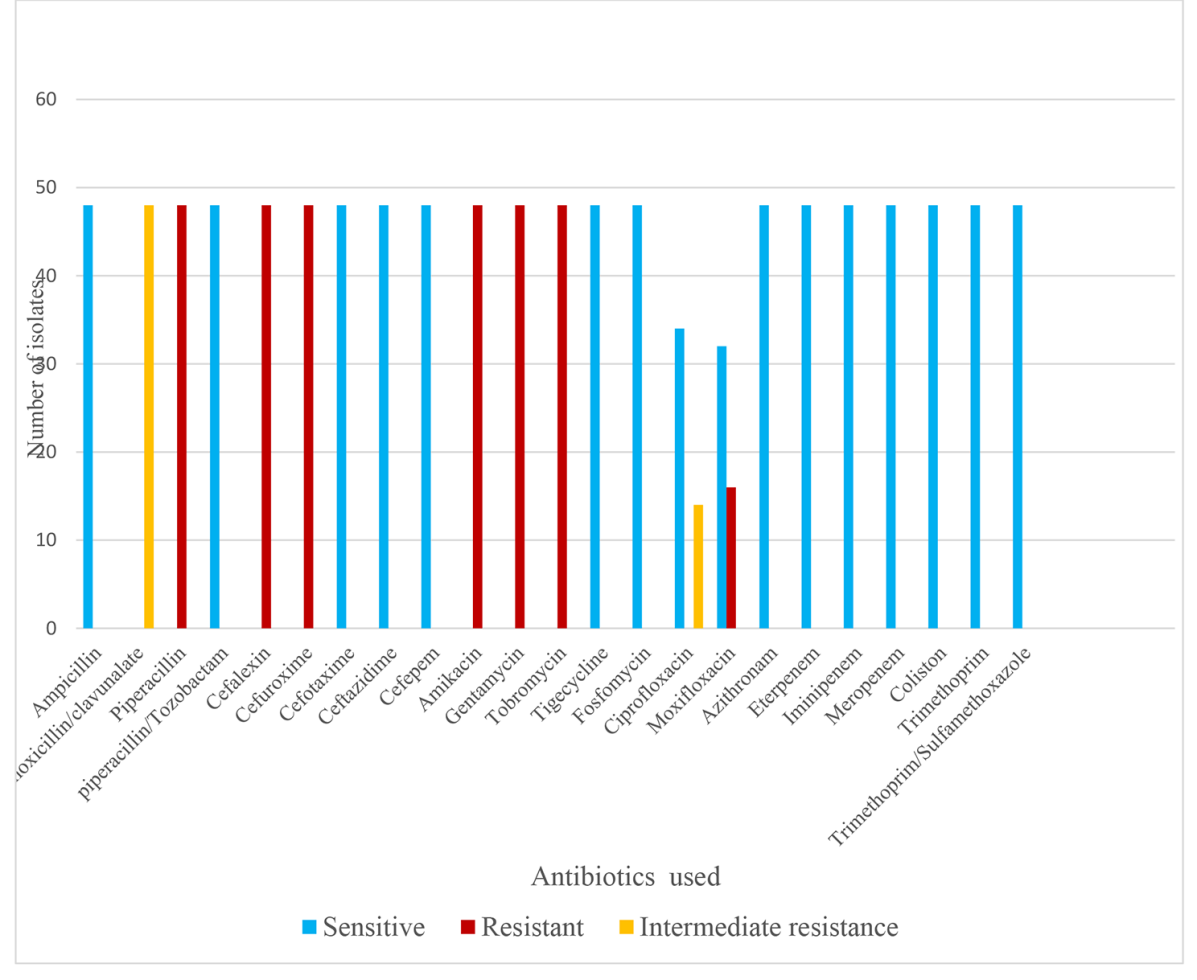
Antibiogram of *Salmonella enterica* serovars isolated from different sources in Nigeria in accordance with EUCAST Expert Rules x 3.2

All forty-eight *Salmonella enterica* isolates were 100% susceptible to ampicillin, piperacillin-tazobactam, cefotaxime, ceftazidime, cefepime, azithronam, ertapenem, imipenem, meropenem, tigecycline, fosfomycin, colistin, trimethoprim, and trimethoprim-sulfamethoxazole. Meanwhile, 16 (33.33%) isolates were resistant to moxifloxacin and 14 (29.2%) isolates showed intermediate resistance to Ciprofloxacin. There was no phenotypic expression of extended-spectrum β-lactamase (ESβL), inducible AmpC, Metallo- β-Lactamase, or *bla*OXA-48 among the isolates **(**Fig. 2**).**

All isolates contained intrinsic chromosomal encoded aminoglycoside acetyltransferase aac(6)-Iaa resistance genes as well as mdf(A) genes coding for a multidrug efflux pump. Acquired quinolone resistance gene *qnr*B was detected in four strains of *Salmonella* Give 8.3% (4/48), while 12 strains of *Salmonella* Give harbored *qnr*B19. All *Salmonella* serovars harbor efflux mechanism genes *sinH, mdsB*, and *mdsA* and genes *golT* and *golS* coding for resistance to copper/gold and gold, respectively. However, genes *pcoE, pcoS, pocR, pcoD, pcoC, pcoB, pcoA, silP, silA, silB, silF, silC, silR, silS, and silE* coding for resistance to copper, silver, and copper/silver were found in only one *Salmonella* Senftenberg strain. Plasmid replicons were detected in 20 of the 48 isolates. All 16 *Salmonella* Give strains harbored plasmid Col440I_1, while plasmid incFIB.B/incFII.S was detected in four *Salmonella* Typhimurium strains **(**Table 2**).**

**Table 2:** Summary of the intrinsic and acquired resistance genes, transporter genes and plasmid replicons in 48 Salmonella enterica strains from Nigeria

Strain ID	Serotype	Source	Intrinsic genes	Acquired genes/Transporters	Plasmid replicon
19CS0255	Senftenberg	Animal (cattle)	*aac.6…Iaa/mdf(A)*	sinH, mdsB. mdsA, golT, golS, pcoE,pcoS, pocR, pcoD, pcoC, pcoB, pcoA,silP,silA,silB,silF,silC,silR,silS,silE	-
19CS0257	Abony	Sewage/wastewater	*aac.6…Iaa/mdf(A*)	sinH, mdsB, mdsA, golT, golS	-
19CS0290	Abony	Sewage/wastewater	*aac.6…Iaa/mdf(A)*	sinH, mdsB, mdsA, golT, golS	-
19CS0294	Abony	Human	*aac.6…Iaa/mdf(A)*	sinH, mdsB, mdsA, golT, golS	-
19CS0295	Abony	Human	*aac.6…Iaa/mdf(A)*	sinH, mdsB, mdsA, golT, golS	-
19CS0250	Cotham	Animal (poultry)	*aac.6…Iaa/mdf(A)*	sinH, mdsB, mdsA, golT, golS	-
19CS0263	Cotham	Sewage/wastewater	*aac.6…Iaa/mdf(A)*	sinH, mdsB, mdsA, golT, golS	-
19CS0267	Cotham	Human	*aac.6…Iaa/mdf(A)*	sinH, mdsB, mdsA, golT, golS	-
19CS0269	Cotham	Human	*aac.6…Iaa/mdf(A)*	sinH, mdsB, mdsA, golT, golS	-
19CS0271	Cotham	Human	*aac.6…Iaa/mdf(A)*	sinH, mdsB, mdsA, golT, golS	-
19CS0272	Cotham	Animal (cattle)	*aac.6…Iaa/mdf(A)*	sinH, mdsB, mdsA, golT, golS	-
19CS0274	Cotham	Sewage/wastewater	*aac.6…Iaa/mdf(A)*	sinH, mdsB, mdsA, golT, golS	-
19CS0275	Cotham	Human	*aac.6…Iaa/mdf(A)*	sinH, mdsB, mdsA, golT, golS	-
19CS0277	Cotham	Sewage/wastewater	*aac.6…Iaa/mdf(A)*	sinH, mdsB, mdsA, golT, golS	-
19CS0278	Cotham	Human	*aac.6…Iaa/mdf(A*)	sinH, mdsB, mdsA, golT, golS	-
19CS0279	Cotham	Human	*aac.6…Iaa/mdf(A)*	sinH, mdsB, mdsA, golT, golS	-
19CS0283	Cotham	Animal (poultry)	*aac.6…Iaa/mdf(A)*	sinH, mdsB, mdsA, golT, golS	-
19CS0284	Cotham	Sewage/wastewater	*aac.6…Iaa/mdf(A)*	sinH, mdsB, mdsA, golT, golS	-
19CS0285	Cotham	Animal (poultry)	*aac.6…Iaa/mdf(A)*	sinH, mdsB, mdsA, golT, golS	-
19CS0288	Cotham	Sewage/wastewater	*aac.6…Iaa/mdf(A*)	sinH, mdsB, mdsA, golT, golS	-
19CS0291	Cotham	Human	*aac.6…Iaa/mdf(A)*	sinH, mdsB, mdsA, golT, golS	-
19CS0292	Cotham	Sewage/wastewater	*aac.6…Iaa/mdf(A)*	sinH, mdsB, mdsA, golT, golS	-
19CS0245	Give	anderes Resistogramm	*aac.6…Iaa/mdf(A*)	qnrB, sinH, mdsB, mdsA, golT, golS	Col440I_1
19CS0246	Give	Animal (cattle)	*aac.6…Iaa/mdf(A)*	qnrB19, sinH, mdsB, mdsA, golT, golS	Col440I_1
19CS0247	Give	Animal (poultry)	*aac.6…Iaa/mdf(A)*	qnrB19, sinH, mdsB, mdsA, golT, golS	Col440I_1
19CS0248	Give	Sewage/wastewater	*aac.6…Iaa/mdf(A)*	qnrB19, sinH, mdsB, mdsA, golT, golS	Col440I_1
19CS0249	Give	Sewage/wastewater	*aac.6…Iaa/mdf(A)*	qnrB19, sinH, mdsB, mdsA, golT, golS	Col440I_1
19CS0251	Give	Animal (poultry)	*aac.6…Iaa/mdf(A)*	qnrB, sinH, mdsB, mdsA, golT, golS	Col440I_1
19CS0259	Give	Animal (cattle)	*aac.6…Iaa/mdf(A)*	qnrB19, sinH, mdsB, mdsA, golT, golS	Col440I_1
19CS0260	Give	Animal (cattle)	*aac.6…Iaa/mdf(A)*	qnrB19, sinH, mdsB, mdsA, golT, golS	Col440I_1
19CS0261	Give	Animal (cattle)	*aac.6…Iaa/mdf(A)*	qnrB, sinH, mdsB, mdsA, golT, golS	Col440I_1
19CS0262	Give	Animal (cattle)	*aac.6…Iaa/mdf(A)*	qnrB19, sinH, mdsB, mdsA, golT, golS	Col440I_1
19CS0264	Give	Human	*aac.6…Iaa/mdf(A)*	qnrB19, sinH, mdsB, mdsA, golT, golS	Col440I_1
19CS0266	Give	Human	*aac.6…Iaa/mdf(A)*	qnrB19, sinH, mdsB, mdsA, golT, golS	Col440I_1
19CS0268	Give	Human	*aac.6…Iaa/mdf(A*)	qnrB19, sinH, mdsB, mdsA, golT, golS	Col440I_1
19CS0280	Give	Human	*aac.6…Iaa/mdf(A)*	qnrB, sinH, mdsB, mdsA, golT, golS	Col440I_1
19CS0289	Give	Human	*aac.6…Iaa/mdf(A)*	qnrB19, sinH, mdsB, mdsA, golT, golS	Col440I_1
19CS0293	Give	Human	*aac.6…Iaa/mdf(A)*	qnrB19, sinH, mdsB, mdsA, golT, golS	Col440I_1
19CS0270	Mokola	Human	*aac.6…Iaa/mdf(A)*	sinH, mdsB, mdsA, golT, golS	-
19CS0273	Mokola	Animal (poultry)	*aac.6…Iaa/mdf(A)*	sinH, mdsB, mdsA, golT, golS	-
19CS0276	Mokola	Human	*aac.6…Iaa/mdf(A)*	sinH, mdsB, mdsA, golT, golS	-
19CS0281	Mokola	Animal (cattle)	*aac.6…Iaa/mdf(A)*	sinH, mdsB, mdsA, golT, golS	-
19CS0282	Mokola	Animal (cattle)	*aac.6…Iaa/mdf(A)*	sinH, mdsB, mdsA, golT, golS	-
19CS0287	Mokola	Sewage/wastewater	*aac.6…Iaa/mdf(A)*	sinH, mdsB, mdsA, golT, golS	-
19CS0253	Typhimurium	Sewage/wastewater	*aac.6…Iaa/mdf(A)*	sinH, mdsB, mdsA, golT, golS	incFIB.B/incFII.S
19CS0256	Typhimurium	Animal (cattle)	*aac.6…Iaa/mdf(A)*	sinH, mdsB, mdsA, golT, golS	incFIB.B/incFII.S
19CS0258	Typhimurium	Human	*aac.6…Iaa/mdf(A)*	sinH, mdsB, mdsA, golT, golS	incFIB.B/incFII.S
19CS0286	Typhimurium	Animal (poultry)	*aac.6…Iaa/mdf(A)*	sinH, mdsB, mdsA, golT, golS	incFIB.B/incFII.S

An average of 100 to 118 virulence gene markers distributed across several *Salmonella* pathogenicity islands (SPIs), clusters, and plasmid operons were detected in all 48 *Salmonella* isolates. A total of 95 virulence genes were common to all *Salmonella* isolates. The virulence genes *ctdB, fae, faeD*, and *faeE* were found only in *Salmonella* Give and *S*. Cotham strains. *IpfA, IpfB, IpfC, IpfD, IpfE, pipB2*, and *sopD2* genes were detected in *Salmonella* Senftenberg, *S*. Abony and *S*. Typhimurium. The virulence genes *spvB, spvC, spvR, ssel, srfH, sseK2*, and *sspH2* were found only in *S.* Typhimurium strains, while the *entE* gene was detected in only *S.* Mokola strains (Table 3). The result of the raw sequence data for the serovar prediction using SISTR and SeqSero indicated a pass QC status for 42 isolates. Only six isolates of serovar Mokola had a warning QC status (Table S1), as only 186 cgMLST330 loci matched the number of cgMLST330 loci found (n = 330). The reason for this warning might be the relatively low number of publicly available genome sequence of serovar Mokola which have beend used for training these tools. In fact, Enterobase, the largest collection of *Salmonella* genome sequences with 403.715 strains (accessed May 2023), contained only three Mokola strains. The multi-locus sequence typing (cMLST), allelic profiles, and sequence types (ST) of 48 *Salmonella enterica* isolates were assigned by comparing the sequences with those in the MLST profile database. The 48 *Salmonella* isolates yielded six unique serovars, and the 7-gene MLST identified one sequence type (ST) for each serovar. Sequence type ST617 is shared by all 17 *Salmonella* Cotham strains while ST516 is shared by all 16 *Salmonella* Give strains. Similar findings were made with the ST19 sequence type for the four *Salmonella* Typhimurium strains and the ST1483 sequence type for the four *Salmonella* Abony strains. The only strain of *Salmonella enterica* subsp. *enterica* serovar Senftenberg belongs to the ST14 sequence type. In the MLST profile database, no sequence type for *Salmonella* Mokola was found **(Table S5).**

**Table 3:** Distribution of *Salmonella* virulence genes clustered within several *Salmonella* pathogenicity Islands (SPIs) and plasmid operons from different source from Nigeria

Strain ID	Serovar	Num of virulence gene	Virulence loci	Virulence genes
19CS0245	Give	100	SPI-1 SPI-2, SPI-3 SPI-11SPI-5, SPI-24/CS54 fimbriae operon	ctdB, fae. faeD faeE sspH1*
19CS0246	Give	100	SPI-1 SPI-11 SPI-2 SPI-3 SPI-5 SPI-24/CS54 fimbriae operon	CtdB fae. faeD faeE sspH1*
19CS0247	Give	100	SPI-1 SPI-11 SPI-2 SPI-3 SPI-5 SPI-24/CS54 fimbriae operon	CtdB fae. faeD faeE sspH1*
19CS0248	Give	100	SPI-1 SPI-11 SPI-2 SPI-3 SPI-5 SPI-24/CS54 fimbriae operon	CtdB fae. faeD faeE sspH1*
19CS0249	Give	100	SPI-1 SPI-11 SPI-2 SPI-3 SPI-5 SPI-24/CS54 fimbriae operon	CtdB fae. faeD faeE sspH1*
19CS0250	Cotham	100	SPI-1 SPI-11 SPI-2 SPI-3 SPI-5 SPI-24/CS54 fimbriae operon	CtdB fae. faeD faeE pipB2*
19CS0251	Give	100	SPI-1 SPI-11 SPI-2 SPI-3 SPI-5 SPI-24/CS54 fimbriae operon	ctdB fae. faeD faeE sspH1*
19CS0253	Typhimurium	116	SPI-1 Long polar fimbriae cluster, Plasmid encoded fimbriae cluster, Plasmid virulence Operon SPI-2 SPI-3 SPI-5 SPI-12 SPI-24/CS54 fimbriae operon	gogB grvA ipfA ipfB ipfC ipfD ipfE pefA pefB pefC pefD pipB2 rck sodC1 sopD2 spvB spvC spvR ssel.srfH sseK2 sspH2*
19CS0255	Senftenberg	101	SPI-1 Long polar fimbriae cluster SPI-3 SPI-5	ipfA ipfB ipfC ipfD ipfE pipB2 sopD2*
19CS0256	Typhimurium	116	SPI-1 SPI-2 SPI-3 Long polar fimbriae cluster, Plasmid encoded fimbriae cluster, Plasmid virulence Operon SPI-5 SPI-12 SPI-24/CS54 fimbriae operon	gogB grvA ipfA ipfB ipfC ipfD ipfE pefA pefB pefC pefD pipB2 rck sodC1 sopD2 spvB spvC spvR ssel.srfH sseK2 sspH2*
19CS0257	Abony	106	SPI-1 SPI-2 SPI-3 Long polar fimbriae cluster SPI-5 SPI-12 SPI-24/CS54 fimbriae operon	ipfA ipfB ipfC ipfD ipfE pipB2 shdA sodC1 sopD2 sseK2 sspH2*
19CS0258	Typhimurium	118	SPI-1 SPI-2 SPI-12 SPI-24/CS54 Long polar fimbriae cluster, Plasmid encoded fimbriae SPI-5iae cluster, Plasmid virulence Operon fimbriae operon	gogB grvA ipfA ipfB ipfC ipfD ipfE pefA pefB pefC pefD pipB2 rck shdA sodC1 sopD2 spvB spvC spvR ssel.srfH sseK2 sspH2 sspH1*
19CS0259	Give	100	SPI-1 SPI-11 SPI-2 SPI-3 SPI-5 SPI-24/CS54 fimbriae operon	ctdB fae. faeD faeE sspH1*
19CS0260	Give	100	SPI-1 SPI-11 SPI-2 SPI-3 SPI-5 SPI-24/CS54 fimbriae operon	ctdB fae. faeD faeE sspH1*
19CS0261	Give	100	SPI-1 SPI-11 SPI-2 SPI-3 SPI-5 SPI-24/CS54 fimbriae operon	ctdB fae. faeD faeE sspH1*
19CS0262	Give	100	SPI-1 SPI-11 SPI-2 SPI-3 SPI-5 SPI-24/CS54 fimbriae operon	CtdB fae. faeD faeE sspH1*
19CS0263	Cotham	100	SPI-1 SPI-2 SPI-11 SPI-3 SPI-5 SPI-24/CS54 fimbriae operon	ctdB fae. faeD faeE pipB2*
19CS0264	Give	100	SPI-1 SPI-11 SPI-2 SPI-3 SPI-5 SPI-24/CS54 fimbriae operon	CtdB fae. faeD faeE sspH1*
19CS0266	Give	100	SPI-1 SPI-11 SPI-2 SPI-3 SPI-5 SPI-24/CS54 fimbriae operon	ctdB fae. faeD faeE sspH1*
19CS0267	Cotham	100	SPI-1 SPI-2 SPI-11 SPI-3 SPI-5 SPI-24/CS54 fimbriae operon	ctdB fae. faeD faeE pipB2*
19CS0268	Give	100	SPI-1 SPI-11 SPI-2 SPI-3 SPI-5 SPI-24/CS54 fimbriae operon	ctdB fae. faeD faeE sspH1*
19CS0269	Cotham	100	SPI-1 SPI-2 SPI-11 SPI-3 SPI-5 SPI-24/CS54 fimbriae operon	ctdB fae. faeD faeE pipB2*
19CS0270	Mokola	102	SPI-1 SPI-2 SPI-3 SPI-5 SPI-12 SPI-24/CS54 fimbiae operon	entE pipB2 shdA sodC1 sopD2 sseK2 sspH2*
19CS0271	Cotham	100	SPI-1 SPI-2 SPI-11 SPI-3 SPI-5 SPI-24/CS54 fimbriae operon	ctdB fae. faeD faeE pipB2*
19CS0272	Cotham	100	SPI-1 SPI-2 SPI-11 SPI-3 SP1-5 SPI-24/CS54 fimbriae operon	ctdB fae. faeD faeE pipB2*
19CS0273	Mokola	102	SPI-1 SPI-2 SPI-3 SPI-5 SPI-12 SPI-24/CS54 fimbriae operon	entE pipB2 shdA sodC1 sopD2 sseK2 sspH2*
19CS0274	Cotham	100	SPI-1 SPI-2SPI-11 SPI-3 SPI-5 SPI-24/CS54 fimbriae operon	ctdB fae. faeD faeE pipB2*
19CS0275	Cotham	100	SPI-1 SPI-2SPI-11 SPI-3 SPI-5 f SPI-24/CS54 imbriae operon	ctdB fae. faeD faeE pipB2*
19CS0276	Mokola	102	SPI-1 SPI-2 SPI-3 SPI-5 SPI-12 SPI-24/CS54 fimbriae operon	entE pipB2 shdA sodC1 sopD2 sseK2 sspH2*
19CS0277	Cotham	100	SPI-1 SPI-2SPI-11 SPI-3 SPI-5 SPI-24/CS54 fimbriae operon	ctdB fae. faeD faeE pipB2*
19CS0278	Cotham	100	SPI-1 SPI-2 SPI-11 SPI-3 SPI-5 SPI-24/CS54 fimbriae operon	ctdB fae. faeD faeE pipB2*
19CS0279	Cotham	100	SPI-1 SPI-2 SPI-11 SPI-3 SPI-5 SPI-24/CS54 fimbriae operon	ctdB fae. faeD faeE pipB2*
19CS0280	Give	100	SPI-1 SPI-2SPI-11 SPI-3 SPI-5 SPI-24/CS54 fimbriae operon	ctdB fae. faeD faeE sspH1*
19CS0281	Mokola	100	SPI-1 SPI-2 SPI-3 SPI-5 SPI-24/CS54 fimbriae operon	entE pipB2 shdA sodC1 sopD2*
19CS0282	Mokola	**102**	SPI-1 SPI-2 SPI-3 SPI-5 SPI-12 SPI-24/CS54 fimbriae operon	entE pipB2 shdA sodC1 sopD2 sseK2 sspH2*
19CS0283	Cotham	100	SPI-1 SPI-2 SPI-3 SPI-5 SPI-24/CS54 fimbriae operon	ctdB fae. faeD faeE pipB2*
19CS0284	Cotham	100	SPI-1 SPI-2 SPI-3 SPI-5 SPI-24/CS54 fimbriae operon	ctdB fae. faeD faeE pipB2*
19CS0285	Cotham	100	SPI-1 SPI-2 SPI-3 SPI-5 SPI-24/CS54 fimbriae operon	ctdB fae. faeD faeE pipB2*
19CS0286	Typhimurium	116	SPI-1 SPI- SPI-3 2 Long polar fimbriae cluster, Plasmid encoded fimbriae cluster, Plasmid virulence Operon SPI-5 SPI-12 SPI-24/CS54 fimbriae operon	gogB grvA ipfA ipfB ipfC ipfD ipfE pefA pefB pefC pefD pipB2 rck sodC1 sopD2 spvB spvC spvR ssel.srfH sseK2 sspH2*
19CS0287	Mokola	101	SPI-1 SPI-2 SPI-3 SPI-5 SPI-12 SPI-24/CS54 fimbriae operon	entE pipB2 shdA sodC1 sopD2 sspH2*
19CS0288	Cotham	100	SPI-1 SPI-2 SPI-3 SPI-5 SPI-24/CS54 fimbriae operon	ctdB fae. faeD faeE pipB2*
19CS0289	Give	100	SPI-1 SPI-2 SPI-3 SPI-5 SPI-24/CS54 fimbriae operon	ctdB fae. faeD faeE sodC1 sopD2 sspH1 sspH1*
19CS0290	Abony	106	SPI-1 SPI-2 SPI-3 SPI-24/CS54 Long polar fimbriae cluster SPI-5 fimbriae operon	ipfA ipfB ipfC ipfD ipfE pipB2 shdA sseK2 sspH2*
19CS0291	Cotham	100	SPI-1 SPI-2 SPI-3 SPI-5 SPI-24/CS54 fimbriae operon	ctdB fae. faeD faeE pipB2*
19CS0292	Cotham	100	SPI-1 SPI-2 SPI-3 SPI-5 SPI-24/CS54 fimbriae operon	ctdB fae. faeD faeE pipB2*
19CS0293	Give	100	SPI-1 SPI-2 SPI-3 SPI-5 SPI-24/CS54 fimbriae operon	ctdB fae. faeD faeE sspH1*
19CS0294	Abony	106	SPI-1 SPI-2 SPI-3 SPI-12 SPI-24/CS54 Long polar fimbriae cluster SPI-5 fimbriae operon	ipfA ipfB ipfC ipfD ipfE pipB2 shdA sodC1 sopD2 sseK2 sspH2*
19CS0295	Abony	106	SPI-1 SPI-2 SPI-3 SPI-12 SPI-24/CS54 Long polar fimbriae cluster SPI-5 fimbriae operon	ipfA ipfB ipfC ipfD ipfE pipB2 shdA sodC1 sopD2 sseK2 sspH2*
GCF0000069452ASM694v2	LT2	117	SPI-1 SPI-2 SPI-3 SPI-5 SPI-12 SPI-24/CS54 Long polar fimbriae cluster, Plasmid encoded fimbriae cluster, Plasmid virulence Operon fimbriae operon	gogB grvA ipfA ipfB ipfC ipfD ipfE pefA pefB pefC pefD pipB2 rck sodC1 sopD2 SshdA spvB spvC spvR ssel.srfH sseK2 sspH2*

*Represents the following 95 virulence genes: avrA csgA csgB csgC csgD csgE csgF csgG entA entB fepC fepG fimC fimD fimF fimH fimI invA invB invC invE invF invG invH invI invJ mgtB mgtC mig.14 misL ompA orgA orgB orgC pipB prgH prgI prgJ prgK ratB sicA sicP sifA sifB sinH sipA.sspA sipB.sspB sipC.sspC sipD slrP sopA sopB.sigD sopD sopE2 spaO spaP spaQ spaR spaS spiC.ssaB sptP ssaC ssaD ssaE ssaG ssaH ssaI ssaJ ssaK ssaL ssaM ssaN ssaO ssaP ssaQ ssaR ssaS ssaT ssaU ssaV sscA sscB sseA sseB sseC sseD sseE sseF sseG sseJ sseK1 sseL steA steB steC

*3° 24’ 23.2128’’ E.* (Longitude)

**Fig. 3:**
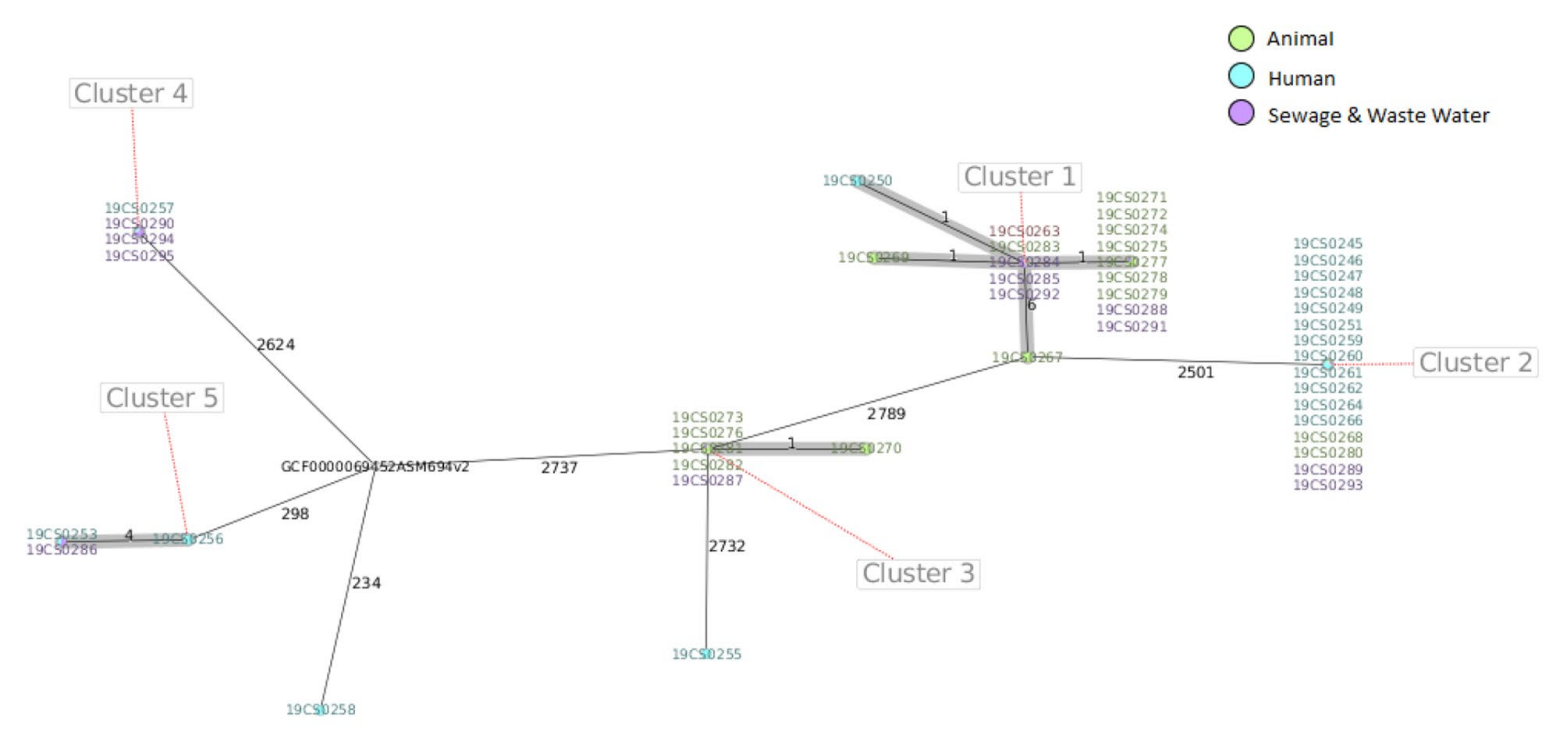
The minimum spanning tree (MST) of *Salmonella* strains (nodes) was used in this study. The node colour corresponds to the source of the strains (see legend). The allelic distances from cgMLST analysis are denoted at branches. Clusters of genetically similar strains were defined using a cut-off of 10 alleles and are visualized in grey

**Fig. 4:**
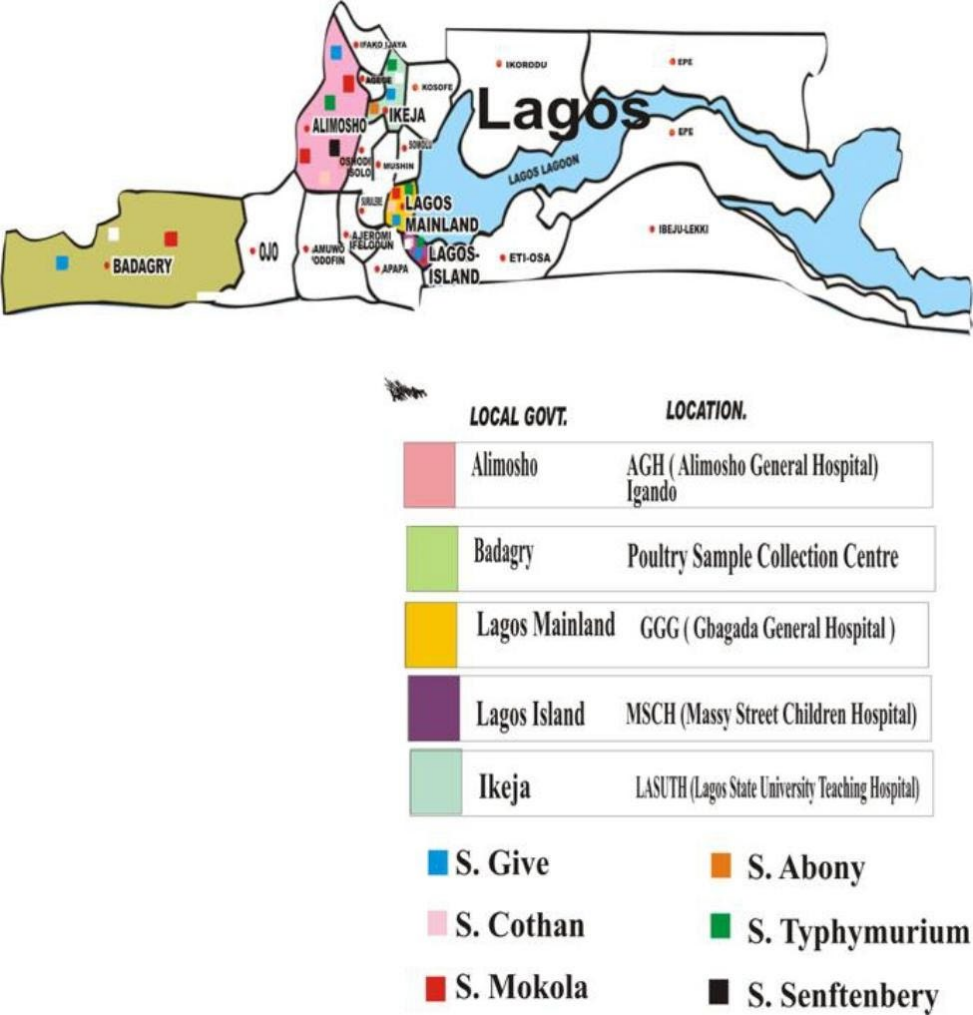
Map of Nigeria showing the location of Lagos State and the distribution of *Salmonella enterica* serovars across different local government areas of Lagos State

The core-genome multi-locus sequence typing (cMLST)clustering of 48 *Salmonella* isolates based on the source of the isolate, type of samples, and clinical diagnosis revealed five distinct clusters of 46 *Salmonella* isolates. Two of the *Salmonella* isolates (*S*. Senftenberg and one *S*. Typhimurium) were not assigned to any cluster. Cluster 1 with ST617 consisted of 17 *Salmonella* strains distributed among human isolates (2 strains), animal isolates (7 strains from cattle and 3 strains from poultry), and five environmental isolates. Cluster 2 with ST516 included 16 *Salmonella* strains, i.e., 12 human isolates, 2 animal isolates (one cattle and one poultry), and 2 environmental isolates. Cluster 3 with unknown ST consisted of 6 strains, of which 5 strains were from animals (three strains from poultry and two from cattle) and one strain from an environmental source. Cluster 4 with ST1483 comprised 4 strains, of which 3 were from environmental samples and one was from a human patient. Cluster 5 with ST19 was made up of 3 strains, of which 2 were from human patients and one from animal sources (**Table S6** and Fig. 3. The distribution of the isolates within the local government areas is shown in Fig. 4.

## Discussion

The intensity with which non-typhoidal *Salmonella* (NTS), responsible for foodborne diseases, acquires antimicrobial resistance genes over the years is worrisome and of public health concern [50]. Using WGS and bioinformatics tools, this study examined clonal relationships among NTS isolates from humans, animals, and the environment, their virulence potential, and the presence of antimicrobial resistance genes that may pose a public health threat if spread to other bacterial agents. Two thousand five hundred twenty-two samples yielded 48 different *Salmonella*. The infection was present in 0.9% (19/2002) of human samples, 4% (17/420) of animal samples, and 12% (12/100) of environmental sample (sewage and wastewater).

The 4% prevalence rate from animal sources (poultry: 7.3% (11/150) and cattle: 2.2% (6/270)) reported in this study is lower than that reported by Jibril et al. [51] with 14.3% for animal faecal samples. A similar high prevalence was documented in other African countries, such as Ethiopia with 14.9% [52] and Ghana with 44% [53]. This difference could be caused by sample size, as a much lower number of samples was collected in this study. In Denmark, an annual report on *Salmonella enterica* zoonosis revealed a prevalence of 0–1.8% [54]. The low prevalence recorded in Denmark has been associated with effective surveillance and control programs, a situation that is not well-footed in Nigeria.

The dominant serotype in this study was *S*. Cotham (35.4%), followed by *S*. Give (33.3%), *S*. Mokola (12.5%), *S*. Typhimurium (8.3%), *S*. Abony (8.3%), and *S*. Senftenberg (2.1%). Except for S. Typhimurium, the serovars found in this study had not been characterized in Nigeria before, either from animals (poultry and cattle), humans, or the environment. Besides, little is known about their potential to cause human disease. Interestingly, serovars *S*. Give and *S*. Cotham were cultured from patients, animals, and wastewater from abattoirs and the hospital environment. Thus, both serovars (*S*. Give and *S*. Cotham) may be emerging *Salmonella* serovars in Nigeria. Although some of the serovars isolated in this study are not commonly associated with human salmonellosis, they may be of special importance in the Nigerian setting. S. Give, for example, was reported in a multistate outbreak in Germany in 2004 that resulted in severe gastroenteritis and hospitalization of those infected [55].*S.* Give has become widespread recently in poultry in Nigeria [51] and Burkina Faso [56], and serovars S. Give and *S*. Cotham were reported repeatedly from animal sources in Nigeria in the past [11, 57]. This finding needs further research for a reliable risk assessment. In sub-Saharan Africa, *S.* Typhimurium and *S.* Enteritidis strains were the prevailing iNTS serovars associated with invasive systemic infections in children and adults [58, 59], with an estimated mortality rate among in-patients ranging from 4.4 to 27% for children [60] and 22 to 47% for adults [61]. In Burkina Faso, *Salmonella* Enteritidis-associated bacteremia was also documented [56]. In this study, seven strains of *S*. Give, one each of *S*. Typhimurium, and *S*. Senftenberg strain, were iNTS. Although a previous study in Nigeria documented *S.* Typhimurium and *S.* Enteritidis-associated bacteremia [62], these are the first reported iNTS Give and Senftenberg-associated bacteremia cases in Nigeria. The emerging iNTS *S*. Typhimurium and *S* Enteritidis seem to be already prominent in Nigeria, and their potential to cause human disease and spread to other neighboring countries is not in doubt.

All forty-eight *Salmonella enterica* isolates were 100% susceptible to piperacillin-tazobactam, cefotaxime, ceftazidime, cefepime, azithronam, ertapenem, imipenem, meropenem, tigecycline, fosfomycin, colistin, trimethoprim, and trimethoprim-sulfamethoxazole.

The results also revealed that none of the *Salmonella* isolates produced extended-spectrum β-lactamases (ESβL), Metallo-β-lactamases, AmpC, or OXA-48 phenotypically.

Chromosomally encoded aminoglycoside acetyltransferase *aac.(6)-Iaa* resistance genes were present in all 48 *Salmonella* isolates (100%) and conferred resistance to aminoglycosides like tobramycin, amikacin, and gentamicin, which are known to bind the bacterial 50 S subunit of the ribosome and prevent protein synthesis. The presence of this gene has made the use of aminoglycoside ineffective in vivo especially for the treatment of invasive Salmonellosis and as such EUCAST expert rule 3.2 of 2019 reported all aminoglycoside should be reported as resistant irrespective of the result of the susceptibility results.

Acquired quinolone resistance genes were detected in all 16 (100%) strains of *S.* Give. Plasmid-mediated quinolone resistance (PMQR) *qnrB* gene was detected in 4, while the *qnrB19* gene was found in 12 *S*. Give strains. All *S*. Give strains harbored plasmid Col440I. Fifteen S. Give strains (93.75%) were resistant to ciprofloxacin and 16 (100%) to moxifloxacin. These strains were also phenotypically resistant to this antibiotic family but susceptible to all other tested antibiotics. This result agrees with the results of a recent report on phenotypic and genotypic antimicrobial resistance and the presence of resistance genes in *Salmonella* isolates from poultry, where neither phenotypic expression of ESBL nor ESBL genes were present in the *Salmonella* strains studied [51]. This observation corresponds well with the fact that β-lactams are reported to be used rarely or not often in poultry production in Nigeria [63, 64], possibly due to their high price. However, fluoroquinolones have been reported to be used as a growth promoter and possibly contributed to the emergence of *Salmonella* and other bacteria resistant to ofloxacin and ciprofloxacin. Predictions by ResFinder indicated that these *S*. Give strains carried either *qnr*B19 or *qnr*B genes with or without the presence of point mutations in DNA gyrase and topoisomerase. It has been documented that the *qnr*B19 and *qnr*B genes encode transferable fluoroquinolone resistance mechanisms that are responsible for reduced susceptibility to quinolones [65]. Also, a point mutation in the quinolone resistance-determining region (QRDR) of the DNA gyrase-A (*gyr*A) and topoisomerase C (*par*C) genes is known to cause clinical resistance in members of *Enterobacteriaceae* [65]. Similar reports have been documented in *Salmonella* isolates from Nigeria [57, 66, 67]. The high-level detection of *qnr* genes in *S.* Give may contribute not only to the stepwise development of high-level fluoroquinolone resistance but also to its spread between bacteria species [68]. The first report of a plasmid-mediated quinolone resistance (PMQR) mechanism, *qnrA*, was described in the late 1990s, and since then, several variants of the *qnr* gene have been discovered [5].

Among the 48 *Salmonella* isolates in this study, 20 isolates belonging to two serovars contained plasmid replicons. In total, three different plasmid replicas were detected, with Col440I being the most predominant replicon found in the “16” “*S*”. Give was predicted to carry *qnr*B, *qnrB-19, sin*H, *mds*B, *mds*A, *gol*T, and *gol*S, respectively. The Col440II-like plasmid detected in *S.* Schwarzengrund isolates in Chile highlighted the fact that a small pPAB19-4-like plasmid plays an important role in the dissemination of *qnr*B19 [69]. Incompatible plasmids incFIB.B and incFII.S were found in all four strains of *S.* Typhimurium predicted to carry the *sin*H, *mds*B, *mds*A, *gol*T, and *gol*S genes. The plasmids incFIB.B and incFII.S from *S*. Typhimurium were aligned with the reference sequence GCF0000069452ASM694v2 (LT2). There was 100% identity with 100.0% query cover between the sequences in this study and the reference sequence.

In this study, acquired *golT* genes coding for resistance to copper or gold and *golS* genes coding for resistance to gold were detected in all 48 isolates, with a percentage of similarity ranging from 96.71 to 100% when compared to the GCF0000069452ASM694v2 (LT2) reference strain. Furthermore, the only iNTS *S*. Senftenberg strain detected in this study harbored several genes that confer resistance to heavy metals such as copper (*pcoE, pcoS, pocR, pcoD, pcoC, pcoB*, and *pcoA*), copper/silver (*silA, silB, silF, silC, silR*, and *silS)*, and silver (*silP* and *silE*). The presence of these heavy metal resistance genes in this strain and its role as an invasive pathogen remain subjects of concern.

Several virulence gene markers of *Salmonella enterica* distributed across several *Salmonella* pathogenicity islands (SPIs) have been found responsible for systemic transmission leading to severe infections [70, 71]. Virulence genes found in all 48 *Salmonella* isolates were predicted by all-vfbd.xls. 100 to 118 virulence gene markers distributed across several *Salmonella* pathogenicity islands (SPIs), clusters, prophages, and plasmid operons were found in each isolate. A total of 95 virulence genes were predicted to be common to all six *Salmonella enterica* serovars. The virulence genes *ctdB, fae, faeD*, and *faeE* were common to *Salmonella* Give, and *S*. Cotham. While *ipfA, ipfB, ipfC, ipfD, ipfE, pipB2*, and *sopD2* were detected in *S.* Senftenberg, *S*. Abony, and *S*. Typhimurium. The virulence genes spvB, spvC, spvR, ssel.srfH, sseK2, and sspH2 were found only in *S.* Typhimurium, while the *entE* was unique to *S.* Mokola. However, *rck* genes that confer resistance to or protect against complement-mediated immune response were detected in all *S*. Typhimurium strains with 100% homology to the gene of strain GCF0000069452ASM694v2 (LT2).

In *Salmonella* pathogenicity, the type 3 secretion system (T3SS), encoded by SPI-1 and SPI-2, contains major virulence determinants. The presence of the major virulence factors *avr*A, *mgt*C, *sop*B, *ssa*Q, and *inv*A in all 48 isolates is an indication of their ability to colonize the liver of the host [50] and therefore may cause serious human disease. The ssp.H1 and *ste*B genes coding for effectors are found in all 16 (100%) *S*. Give strains and one (25%) *S*. Typhimurium strain. These effector genes are mediators of cell invasion and modifications, which are major contributing factors to intracellular growth [72]. The cytolethal distending toxin islet gene (cdtB) was detected in all the *S*. Give and *S*. Cotham in this study. This gene has been reported to play a vital role in disease pathogenesis. This toxin islet has been known to cause DNA damage and cell cycle arrest in impaired cells [73]. The islet gene (cdtB) was detected in *Salmonella* Telelkebir in a similar study conducted in Southwestern Nigeria [27].

The *sop*A and *sop*E2 pseudogenes were detected in all 48 isolates. *SopD2* pseudogenes were found in all *S*. Abony, *S*. Typhimurium, *S*. Senftenberg, and *S.* Mokola isolates, while *shd*A pseudogenes were confined to *S*. Abony and *S.* Mokola isolates. Langridge et al. [74] defined a pseudogene as a “gene with a mutation” (i.e., a premature stop codon, frameshift, truncation, or syntenic deletion) compared to an intact version of that gene. It is easier for *Salmonella* to enter epithelial cells when the effector genes found in *Salmonella* pathogenicity island 1 (SPI-1) are pseudogenized. This enhances *Salmonella* adaptability to systemic infection in humans [75, 76].

As part of the limitations of the study, it was not possible to determine if the number of pseudogenes present in each of the 48 *Salmonella enterica* strains was due to identical or non-identical mutations. The *Salmonella* plasmid virulence (*spv*) locus harbors five genes designated *spv RABCD. The Salmonella* virulence plasmid (*spv*)-*RBC* was found in the four *S*. Typhimurium strains. The expression of the *spv* genes has been reported to play a role in the intracellular multiplication of *Salmonellae* by distorting the cytoskeleton of the eukaryotic cells using its *spv*B ADP-ribosylates actin [77, 78]. The two operons of the chaperone–usher class *pef* and *ipf* (plasmid-encoded fimbriae) known to mediate adhesion of *S.* Typhimurium to the small intestine in mice were detected and confined to four *S*. Typhimurium strains (*pefA, pefB, pefC*, and *pefD*), while the long polar fimbriae operons (*ipfA, ipfB, ipfC*, and *ipfD*) were detected in four *S*. Abony and four *S*. Typhimurium. Other fimbriae operons of the chaperon class detected contain *fimC, fimD, fimF, fimH, fimI, steA, steB*, and *steC*, which are well conserved in all 48 *Salmonella* isolates. The four *Salmonella* invasion proteins (SipsABCD), which have been shown to play essential roles in the secretion and translocation of SPI-1 effectors, are present in all isolates [79].

The *in silico* serotype prediction with Seqsero in comparison to the serotyping scheme passed the Fast-QC threshold, with the QC status indicating “PASS” for 5 serotypes except for serotype *S*. Mokola (6 isolates) showing “WARN QC” status for the quality of the sequences. There was no ST number available for *S*. Mokola from the MLST profile database. This is an indication that the complete genomic sequence of serovar *S*. Mokola has not been deposited in the global *Salmonella* database yet. This result represents the first complete genomic sequence of *S*. Mokola serovar from Nigeria.

Multilocus sequence typing (MLST), allelic profiles, and sequence types (ST) of the 48 *Salmonella enterica* isolates were assigned by comparing the sequences with those in the MLST profile database. Only one sequence type (ST) each was found for the 17 *S.* Cotham (ST617), 16 *S.* Give (ST516), 4 *S.* Typhimurium (ST19), and 4 *S.* Abony (ST1483), while *S.* Senftenberg (ST14) is the only ST generated from segments of seven housekeeping genes (*aroC, dnaN, hemD, hisD, purE, sucA*, and *thrA*). All seven MLST loci were successfully recovered for the 48 *Salmonella enterica* strains isolated from different sources. The presence of such clones in the samples of human, animal, and environmental origin indicates epidemiological links between STs and reservoir isolates. In Sub-Saharan Africa, NTS and iNTS serovars have been a challenge. The MLST of *S*. Typhimurium is known to be prevalent in Burkina Faso in humans and poultry [80]. Also, *S*. Typhimurium ST313 strain D23580 isolated from a patient with an iNTS infection has been reported from Malawi [81]. *S*. Typhimurium ST19 detected in this study belongs to the globally circulating lineage [82]. It is the dominant ST of the MLST database, which, however, consists of sequences of strains isolated from Europe and Northern America. *S*. Typhimurium ST19 has already caused gastroenteritis in humans [83], which is in accordance with this study as *S*. Typhimurium ST19 was detected in stool samples of patients with diarrheal disease from two study centers 20 km apart. The isolation of *S*. Typhimurium ST19 in hospital wastewater in this study is an indication of its dissemination from clinical sources into the environment. In a related study of WGS analysis of *Salmonella* serovars from animals in North-Central Nigeria, eight diverse sequence types (STs) were detected and the most common STs were ST-321 and ST-19 (n = 4) exhibited by S. Muenster and S. Typhimurium, respectively [26]. The detection of invasive *S*. Typhimurium ST19 clones in the blood of febrile patients with systematic infection has also been reported in Iran [84]. *S*. Typhimurium ST19 has been documented to colonize the gut and cause inflammation by a *Salmonella* pathogenicity island (SPI)-1-mediated process when ingested orally [83]. The MLST of the 48 *Salmonella* isolates based on the source of isolates, type of sample, and clinical prognosis revealed five distinct clusters in the 46 *Salmonella* isolates. Two of the *Salmonella* isolates could not be assigned to any cluster. WGS revealed that strains in each MLST *Salmonella* cluster from this study were closely related and likely shared a common ancestor. The distribution of these clusters within the study areas showed that all MLST clusters, including *S*. Senftenberg, that were not assigned to any cluster were found in the Alimosho local government area (LGA). All of the LGAs chosen for this investigation had isolates in clusters 1 and 2. Blood from a feverish patient at the Lagos State University teaching hospital and stool from a 3-5-year-old with diarrhea at Messy Street Children Hospital both contained invasive iNTS Cotham ST617 in cluster 1. While a genetically identical strain was discovered in wastewater taken from Gbagada general hospital, a few kilometers from Badagry LGA and Alimosho LGA, *Salmonella* Cotham ST617 from the same cluster was also detected in stool samples from poultry birds in Badagry and cattle dung along the Governor Road. Similar to this, S. Give (ST516) strains in cluster 2 were found in the Alimosho LGA from human, animal, and environmental samples. A strain of this serovar was also identified from cattle at Odo-eran abattoir in the same LGA, around 1.6 km distant from Alimosho general hospital. It was noted that the *Salmonella* Give strains that were isolated from wastewater and human samples (stool and blood) were from Alimosho general hospital. Additionally, S. Give (ST516) strains were found in the Lagos Island LGA (Messy Street Children hospital), Badagry LGA (Oko-Afor poultry facility), and Ikeja LGA (place of Lagos University teaching hospital). The discovery of this disease in these three LGAs illustrates the spread and circulation of ST516 within Lagos environments because they are geographically apart. In Nigeria, *S*. Give and *S*. Cotham serotypes have recently been reported in water, fecal samples feed, dust, and boot swabs from different poultry farms within the same and different localities [85]. However, local environmental conditions and a lack of biosecurity measures may have been responsible for the dissemination of similar strains [85]. The presence of the two dominant clones (*S.* Give ST516 and *S.* Cotham ST617) in samples from human, animal, and environmental sources demonstrated the need for further epidemiologic studies to identify the infection and to tailor countermeasures for the local setting. This study points to the fact that transmission of *S.* Give ST516 and *S.* Cotham ST617 may occur not only from person to person but also from animal to human. Controlling environmental contamination and potential control methods that could serve as a guide for appropriate waste management require special consideration. Near Alimosho General Hospital are two large, overburdened garbage dump sites, one of which is only 300 m away and the other is 1.3 km distant. The area is occupied by low-income, lower, and upper-middle-class residents, but no functional waste disposal treatment unit is available. These dump sites have grossly polluted the environment, including the groundwater [86]. This may have contributed to the high prevalence of *Salmonella* infection within that area and continuous spread to other areas of the state.

## Conclusion

The characterization of *Salmonella* isolates from hospital, environmental, and animal production sources using different molecular tools was conducted. The study revealed six serotypes, with *S*. Give and *S*. Cotham as the most predominant ones. Closely related *Salmonella* clones were detected in these samples, pointing to an epidemiological link between serotypes and sequence types. The study also showed that the isolates were resistant to multiple antibiotics, as reflected by the presence of intrinsic and acquired genes conferring resistance to antibiotics and heavy metals. A wide range of virulence genes that help the organism become pathogenic in the host were detected from the whole genomic sequences. It becomes obvious that it is time to act and develop a strategy for Nigeria against the spread of some emerging NTS *Salmonella* serovars, such as *S.* Give and *S*. Cotham. This strategy must take into consideration food-producing animals, i.e., occupational animal contacts and food contamination, spill-back risk of the already heavily polluted environment, which arises from indiscriminate disposal of refuse and untreated wastewater discharge (effluent) from the hospital environment, and/or other sources, and consumer behavior and food availability. For the One Health context, these results are alarming, and the risk of further spread of plasmid replicon Col440I_1 and virulence genes is imminent if no immediate action is taken. The first step must be the control and prevention of the evolution of new and more virulent NTS in hospitals and veterinary clinics. Prompt surveillance of emerging pathogens in food chains, proper disposal of refuse, treatment of wastewater from hospitals and food production industries, and control of dump sites near hospitals are essential to preventing outbreaks.

## Electronic supplementary material

Below is the link to the electronic supplementary material.

Supplementary Material 1

## Data Availability

Raw sequences from this study are available and were deposited in the European Nucleotide Archive (ENA) with bio project accession PRJEB56537 in the ENA bio project database: https://www.ebi.ac.uk/ena/browser/view/PRJEB56537.

## Abbreviations

NTS: Non-typhoidal *Salmonella*
INTS: Invasive non-typhoidal *Salmonella*
MLST: Multi-locus sequence typing
CgMLST: Core genome multi-locus sequence typing
CgSNP: Core genome single nucleotide polymorphism
MDR: Multiple-drug resistant
EsβL: Extended spectrum beta-lactamases
KPC: *Klebsiella pneumoniae* producing carbapenemase
MBL: Metallo-beta-lactamase
ST: sequence type
MT: Metric tons
NAFDAC: National Agency for Food and Drug Administration and Control.
PMQR: plasmid-mediated quinolone resistance
SPI: *Salmonella* Pathogenicity Island
LGA: Local Government Area
RFLP: Restriction fragment length polymorphism
VNTR: Variable number of tandem repeats
MLVA: Multiple Locus Variable-Number Tandem Repeat Analysis
MALDI-TOF MS: matrix-assisted laser desorption/ionization-time of flight mass spectrometry
